# Increased signalling of EGFR and IGF1R, and deregulation of PTEN/PI3K/Akt pathway are related with trastuzumab resistance in HER2 breast carcinomas

**DOI:** 10.1038/bjc.2012.85

**Published:** 2012-03-27

**Authors:** A Gallardo, E Lerma, D Escuin, A Tibau, J Muñoz, B Ojeda, A Barnadas, E Adrover, L Sánchez-Tejada, D Giner, F Ortiz-Martínez, G Peiró

**Affiliations:** 1 Department of Pathology, Hospital de la Santa Creu i Sant Pau, Autonomous University of Barcelona, Avda. Sant Antoni Ma Claret 167, 08025, Barcelona, Spain; 2 Department of Clinical Oncology, Institut de Recerca, Hospital de la Santa Creu i Sant Pau, Barcelona, Spain; 3 Department of Clinical Oncology, Hospital de la Santa Creu i Sant Pau, Autonomous University of Barcelona, Barcelona, Spain; 4 Department of Clinical Oncology, Hospital General Universitario, Alicante, Spain; 5 Research Unit, Hospital General Universitario, Alicante, Spain

**Keywords:** breast cancer, HER2, EGFR, IGF1R, PTEN/PI3K/Akt pathway, trastuzumab resistance

## Abstract

**Background:**

Trastuzumab resistance hampers its well-known efficacy to control HER2-positive breast cancer. The involvement of PI3K/Akt pathway in this mechanism is still not definitively confirmed.

**Methods:**

We selected 155 patients treated with trastuzumab after development of metastasis or as adjuvant/neoadjuvant therapy. We performed immunohistochemistry for HER2, ER/PR, epidermal growth factor 1-receptor (EGFR), *α*-insulin-like growth factor 1-receptor (IGF1R), phosphatase and tensin homologue (PTEN), p110*α*, pAkt, pBad, pmTOR, pMAPK, MUC1, Ki67, p53 and p27; mutational analysis of *PIK3CA* and *PTEN*, and *PTEN* promoter hypermethylation.

**Results:**

We found 46% ER/PR-positive tumours, overexpression of EGFR (15%), *α*-IGF1R (25%), p110*α* (19%), pAkt (28%), pBad (22%), pmTOR (23%), pMAPK (24%), MUC1 (80%), PTEN loss (20%), and *PTEN* promoter hypermethylation (20%). *PIK3CA* and *PTEN* mutations were detected in 17% and 26% tumours, respectively. Patients receiving adjuvant trastuzumab with *α*-IGF1R or pBad overexpressing tumours presented shorter progression-free survival (PFS) (all *P*⩽0.043). Also, p110*α* and mTOR overexpression, liver and brain relapses implied poor overall survival (OS) (all *P*⩽0.041). In patients with metastatic disease, decreased PFS correlated with p110*α* expression (*P*=0.024), whereas for OS were the presence of vascular invasion and EGFR expression (*P*⩽0.019; Cox analysis).

**Conclusion:**

Our results support that trastuzumab resistance mechanisms are related with deregulation of PTEN/PI3K/Akt/mTOR pathway, and/or EGFR and IGF1R overexpression in a subset of HER2-positive breast carcinomas.

Breast cancer (BC) is one of the most frequent malignancies in women (Jemal *et al*, 2008). HER2 overexpressing and/or gene amplified tumours represent approximately 25% of all BC, and they are associated with an aggressive phenotype, metastases, resistance to chemotherapy (CT), and poor prognosis (Slamon *et al*, 1987, 1989; Peiro *et al*, 2007; Nguyen *et al*, 2008). Nevertheless, the outcome has changed dramatically with the introduction of trastuzumab, a humanised monoclonal antibody that targets the HER2 extracellular domain (Murphy and Modi, 2009). It is very effective in combination with CT for the treatment of early stages (Viani *et al*, 2007) or metastatic BC (Pegram *et al*, 2004; Brufsky *et al*, 2005), and even as a single-agent for the later group (Vogel *et al*, 2002), showing in both groups of patients a substantial decrease in cancer recurrence and mortality (Slamon *et al*, 2001; Piccart-Gebhart *et al*, 2005; Joensuu *et al*, 2006; Untch *et al*, 2008). Despite its demonstrated clinical benefit, about 30–50% of patients do not respond, and those with metastasis that achieved an initial response to trastuzumab-based regimens will develop drug resistance.

Currently, in clinical practice there are not conclusive biomarkers that allow the selection of patients who will respond to trastuzumab and the exact molecular mechanisms are still not well defined. Several growth factor receptors and signalling molecules have been proposed to be responsible for trastuzumab resistance, such as downregulation of the surface HER2 protein by endocytosis and degradation (Austin *et al*, 2004), p27 downregulation (Lane *et al*, 2001; Nahta *et al*, 2004), activation of insulin-like growth factor 1-receptor (IGF1R) (Lu *et al*, 2001; Nahta *et al*, 2005), interaction between HER2 and epidermal growth factor 1-receptor (EGFR) (Diermeier *et al*, 2005), phosphatase and tensin homologue (PTEN) loss (Nagata *et al*, 2004), phosphoinositide 3-kinase (PI3K)/Akt activation (Esteva *et al*, 2011; Razis *et al*, 2011), MUC1 (Fessler *et al*, 2009) and MUC4 upregulation (Nagy *et al*, 2005), and the crosstalk with the ER signalling pathway (Slamon *et al*, 2001). More recently, the non-receptor tyrosine kinase c-SRC (SRC) has been suggested as a potential key modulator of trastuzumab response (Zhang *et al*, 2011).

Therefore, the aim of our study was to evaluate the relevance of alterations in the PI3K/Akt/mTOR and Ras/mitogen-activated protein kinase (MAPK) signalling pathways, given their role in cell cycle progression. We performed an extensive immunohistochemical and molecular analysis of several biological markers related with these pathways, in a series of patients with HER2-positive BC in stage I-IV, to determine their prognostic relevance, and as a result, their potential involvement in the mechanisms of response to trastuzumab.

## Patients and methods

### Tumour samples and patients’ follow-up

The study was conducted according to the Declaration of Helsinki principles, with approval from the local ethics committees. A total of 155 tumour samples from HER2-positive patients were retrospectively collected from the Department of Pathology of the Hospital de la Santa Creu i Sant Pau (*n*=103) and University General Hospital of Alicante (*n*=52). Patients were staged according to the WHO system, and tumours were histologically graded according to Elston and Ellis method. After pathological diagnosis, patients were treated according to standard protocols. All patients received trastuzumab for the treatment of metastatic disease (*n*=75) after failure of conventional CT with anthracyclines and/or taxanes, or for early stages either adjuvant (*n*=40) or neoadjuvant (*n*=27) therapy. In 13 patients the type of treatment was unknown. Median follow-up was 5.3 years (range 0.17–31 years).

We considered response or non-resistance to trastuzumab treatment when no progression of stable disease occurred. Progression-free survival was defined as the length of time after treatment during which a patient survived with no signs of the disease, and OS as the time to the patients’ death or last follow-up.

### Immunohistochemistry

Tissue microarrays were prepared from paraffin-embedded tissue taken from three representative tumour areas. Sections were stained using the Envision method (Dako, Glostrup, Denmark). HER2 protein and EGFR protein determinations were performed using HercepTest and EGFR pharmaDx (Dako; Glostrup, Denmark), respectively. Antibodies, dilutions, antigen retrieval methods, and suppliers are listed in Table 1. ER/PR and HER2 were evaluated by standard protocols. The EGFR expression was considered positive when complete membrane staining is >10% of tumour cells. The PTEN, pAkt, pBad, p110*α*, p-mTOR, *α*-IGF1R, MUC1, and pMAPK (cytoplasm) scores were calculated by multiplying the percentage of labelled cells by the staining intensity (range 0–300). Loss of PTEN was considered for cutoff scores <75; and overexpression of p110*α*, MUC1, pMAPK, p27 and pAkt were considered for scores ⩾150. Positive *α*-IGF1R and mTOR were considered for scores ⩾220 and ⩾30, respectively. The percentage of stained nuclei was evaluated independently of the intensity for Ki67 (cutoff 20%) and p53 (cutoff 10%). Consensus between three pathologists (AG, EL, and GP) was done for the immunohistochemical results.

### Mutational analysis of *PIK3CA*

Genomic DNA was extracted from frozen tumour or paraffin-embedded tissues and mutational analysis of *PIK3CA* was performed by PCR and direct sequencing using primers for exons 9 and 20 as previously described (Samuels *et al*, 2004).

### Phosphatase and tensin homologue mutation and promoter hypermethylation

Mutational analysis was performed using previously reported PCR conditions and primers for exons 3, 5, 7, and 8 (Bussaglia *et al*, 2000). Methylation-specific PCR was used to assay CpG island methylation status of the *PTEN* promoter gene using the Methylamp One-Step DNA Modification kit (Epigentek, Brooklyn, NY, USA). Three primers sets were used for the PCR as previously reported (Soria *et al*, 2002).

### *In situ* hybridisation analysis

*HER2* gene status was confirmed by fluorescence *in situ* hybridisation (Dako pharmaDx) or chromogenic *in situ* hybridisation (Spot light; Zymed, Paisley, UK) in equivocal cases.

### Statistical analyses

They were performed with the SPSS/win 17. 0 statistical software package (SPSS, Chicago, IL, USA). Qualitative variables were compared with the X2/Fisher tests. A receiver operating characteristic curve and area under the curve were generated to determine a cutoff value of the expression of several biomarkers and the potential clinical utility to predict prognosis. The Kaplan–Meier method and the Cox regression model were used to estimate survival. *P*-values <0.05 were considered statistically significant.

## Results

### Clinicopathological data

The clinicopathological data is summarised in Table 2. Patients were classified into two groups: group A (*n*=75) included patients where trastuzumab was included for treatment of metastatic disease and group B (*n*=67 patients) those with trastuzumab in the adjuvant/neoadjuvant setting. Median age was 55 years (range 31–92 years) and median tumour size was 2.5 cm (range 1–20 cm). Histological grade 1 was seen in 7 (5%) cases, grade 2 in 50 (32%), and grade 3 in 98 (63%) tumours. Vascular invasion was found in 32% (47 of 145) cases. Axillary lymph node dissection was performed in 135 patients, being positive in 89 cases (66%). Tumour stage was IA in 17 (11%) patients, IIA in 29 (18.8%), IIB in 15 (9.7%), IIIA in 42 (27%), IIIB in 18 (11.6%), IIIC in 12 (7.7%), IV in 13 (8.4%), and was unknown in 9 (5.8%) patients. A total of 11 patients were lost in the follow-up, and among those remaining, 56 (39%) were alive with no evidence of disease, 31 (21.5%) alive with disease, and 57 (39.5%) died of the disease (DOD).

### Tumour molecular features

**Figure 1:**
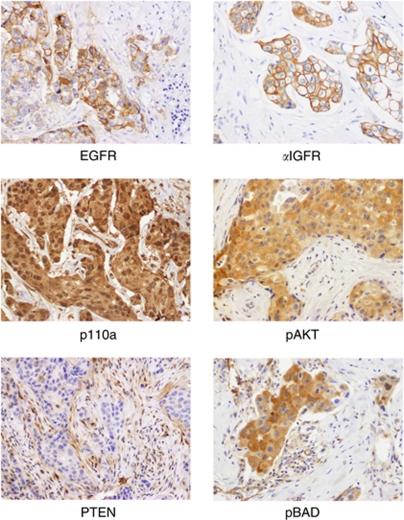
Immunohistochemical expression of EGFR, *α*IGFR, p110a, pAKT, pBad, and loss of PTEN in HER2-positive breast carcinomas.

Table 3 includes the relationship between clinicopathological and molecular data for all patients. Examples of relevant immunohistochemical images are shown in Figure 1.

#### Hormonal receptors (HR)

The HR (either ER or PR) were positive in 46% (67/145) of the tumours, and they were associated with ductal formation (*P*=0.024) and histological grade 1 (*P*=0.048).

#### Growth factor receptors

Increased EGFR expression was found in 15% tumours (21/141) and correlated with high mitotic index (*P*=0.013) and negative HR (*P*<0.000), and there was a trend towards higher tumour grade (*P*=0.061).

Staining of *α*-IGF1R was strong and diffuse (overexpression) in 25% tumours (34/138), in association with high grade (*P*=0.001), high mitotic index (*P*=0.004), and vascular invasion (*P*=0.005).

#### Biomarkers associated with the PI3K/Akt/mTOR and MAPK signalling pathways

Phosphatase and tensin homologue loss was found in 20% of the tumours (30/149), *PTEN* promoter hypermethylation in 20% (22/110) and mutations in 26% (8/30). Phosphatase and tensin homologue loss was associated with vascular invasion (*P*=0.047) and higher grades (*P*=0.065), but neither association with *PTEN* mutation nor hypermethylation was found.

p110*α* (PI3K catalytic subunit) overexpression was present in 19% of the tumours (24/125), and *PIK3CA* somatic missense mutations were identified in 17% (24/142): in exon 20 (nucleotide A3140G, amino acid H1047R) in 15% of the tumours (21/142), whereas mutations in the helical domain of exon 9 (nucleotide G1635C, amino acid E545D) were detected in only 6% (3/50). Interestingly, mutations were present more frequently in tumours with EGFR expression (33% *P*=0.016) and higher nuclear grade (*P*=0.043), but there was no correlation with p110*α* protein expression.

pAkt overexpression was found in 28% of the tumours (40/143) and phosphorylated (inactive) Bad in 22% (30/139) in association with high nuclear (*P*=0.008) and histological grades (*P*=0.001), elevated mitotic index (*P*=0.002), and vascular invasion (*P*=0.006). mTOR overexpression was detected in 23% (33/142) of the tumours, predominantly with high nuclear grade tumours (*P*=0.034), and in association with *α*-IGF1R (47% *P*<0.000), p110*α* (64% *P*=0.028) and pBad (65% *P*=0.027).

Strong pMAPK expression in 24% (22/93) of the tumours predominated in those of low grade (*P*=0.029); MUC1 was overexpressed in 80% of the analysed tumours (77/96) but without significant association with clinicopathological features.

#### Cell proliferation and apoptosis markers

Ki67 >20% was seen in 51% of the tumours (73/144), related with high mitotic index (*P*=0.021). p53 overexpression was found in 30% of the tumours (42/139) associated with HR-negative status (*P*=0.022) and high nuclear grade (*P*=0.009). Only 17% (16/94) of the cases showed p27 nuclear expression but unrelated with any clinical-pathological factors.

### Relationship between biomarkers and recurrence

In all, 61% of the patients developed distant metastases, which were located in the liver (35%), bone (35%), lung (27%), lymph nodes (21%), pleura (18%), central nervous system (CNS; 16%), and skin (14%). Patients with HR-positive tumours presented more frequently bone metastases (*P*=0.008) compared with those with HR-negative status. In contrast, tumours with *α*-IGF1R overexpression rarely metastasised to the liver (*P*=0.009), lung (*P*=0.002), bone (*P*=0.031) or lymph nodes (*P*=0.007). Patients with p110*α*-positive tumours developed more frequently CNS metastases (*P*=0.029). The remaining proteins studied here showed a trend or were not associated with any specific site of dissemination (see Table 4).

### Survival analyses

In order to perform the survival analysis in similar patients groups, we excluded those that received neoadjuvant CT or stage IV. Therefore, 51 patients remained in group A and 38 patients in group B. Supplementary Figures 1 and 2 include the Kaplan–Meier curves for both groups. Table 5 shows the results of the multivariate analysis.

#### Metastatic BC (group A)

A total of 47 patients (92%, 47/51) had tumour recurrence with a median PFS of 2.6 years (range 1.01 to 11.64 years) and 65% (33/51) of the patients DOD with a median OS of 7.5 years (range 0.17 to 21 years).

Univariate analysis (Kaplan–Meier; log rank test) showed that shorter PFS was associated with vascular invasion (*P*=0.042), mutated *PTEN* (*P*=0.045), EGFR (*P*=0.026), p110*α* (*P*=0.004), pAkt overexpression (*P*=0.016), and CNS metastases (*P*=0.002). Poor OS correlated with positive lymph node status (*P*=0.013), EGFR (*P*=0.006), p110*α* (*P*=0.079), pAkt overexpression (*P*=0.042), tumour stage (*P*=0.003), and tumour relapses in the liver (*P*=0.059) or in CNS (*P*=0.005).

Multivariate analyses for PFS revealed that only the presence of metastases to the CNS (*P*=0.020, HR 3.59, CI 1.23–10.51) and p110*α* overexpression (*P*=0.024, HR 2.75, CI 1.14–6.49) emerged as significant predictors of relapse. Worse OS was seen for vascular invasion (*P*=0.015, HR 3.36; CI 1.22–8.94), EGFR expression (*P*=0.019, HR 5.25; CI 1.32–20.92), and metastases to the CNS (*P*=0.009, HR 4.22; CI 1.44–12.38) (Cox regression model).

#### Early-stage BC (group B)

Only 11% (4/38) of the patients had tumour recurrence and 5% died from the tumour. These events might be, however, related to the short follow-up of the majority of the patients (median 2.82 years). Median PFS was 2.81 years (range 1.00–8.28 years) and for OS was 2.82 years (range 1.00–8.42 years).

Shorter PFS was associated with *α*-IGF1R (*P*=0.028), pBad overexpression (*P*=0.003), and tumour recurrence in the liver (*P*=0.003) or the bone (*P*=0.001). Poor OS correlated with high tumour grade (*P*<0.000), overexpression of p110*α* (*P*=0.041) and mTOR (77 *vs* 100% in negative cases; *P*=0.006), and tumour recurrence in the liver (*P*=0.009) and CNS (*P*=0.011) (Kaplan–Meier; log rank test). Nevertheless, the multivariate analysis (Cox regression) showed that these results did not reach any statistically significance, probably due to the small number of events in this group.

## Discussion

In the current study, we performed an extensive immunohistochemical and molecular analysis of biological markers related with the PI3K/Akt/mTOR and Ras/MAPK signalling pathways in a series of HER2-positive BC patients who received trastuzumab for metastatic disease or as first-line therapy in earlier stages. We found that patients with primary tumours showing alterations in EGFR and PTEN/PI3K/Akt had shorter PFS and OS despite trastuzumab treatment when given at advanced stage (metastatic disease), supporting their role in the mechanisms of response. Our results in the group of patients in earlier stages who received trastuzumab as adjuvant/neoadjuvant therapy demonstrated that those having tumours with IGF1R overexpression and inactive Bad had shorter PFS. Poorer OS was seen in patients who developed metastatic disease especially in the brain and liver, and with p110*α* and mTOR overexpressing tumours. Nevertheless, none of the factors had an independent prognostic value, probably related with the small number of events and short follow-up of this group.

PI3K/Akt signalling is one of the most critical cancer-promoting pathways through upregulation of growth factor receptors (EGFR, IGF1R, HER2, etc) or PTEN inactivation (Lu *et al*, 1999) and recently considered a major determinant of trastuzumab resistance (Nagata *et al*, 2004; Berns *et al*, 2007; Esteva *et al*, 2011; Razis *et al*, 2011). HER2 and EGFR coexpression has a considerable inhibitory effect on this drug (Diermeier *et al*, 2005) and are associated with poor prognostic factors such as high grade, negative HR status, and vascular invasion (Abd El-Rehim *et al*, 2004). In agreement with these findings, we found coexpression in 15%, which in turn was related with *PIK3CA* mutations. Insulin-like growth factor 1-receptor has an important role in growth and invasiveness of BC (Peiro *et al*, 2009, 2011) and recently has also been involved in trastuzumab resistance (Lu *et al*, 2001; Nahta *et al*, 2005; Harris *et al*, 2007). In fact, we found IGF1R overexpression in 25% of the tumours, especially in those of early stage patients who developed recurrences. Of note, there is considerable evidence that both IGF1R and EGFR crosstalk in BC cells and their coactivation occurs in approximately 25% of BC, related with poor outcome (Harris *et al*, 2001, 2007; Lu *et al*, 2001; Abd El-Rehim *et al*, 2004). Therefore, it would be expected that those patients would be more likely to be resistant to trastuzumab.

*PTEN* encodes a protein that inhibits activation of the PI3K/Akt/mTOR signalling pathway (Panigrahi *et al*, 2004). The PTEN inactivation may be related with gene mutations (<5% of sporadic BC) (Vanhaesebroeck and Alessi, 2000) or promoter hypermethylation (20%) (Saal *et al*, 2005), resulting in PTEN loss that occurs in about half of the tumours (Nagata *et al*, 2004; Lerma *et al*, 2008; Esteva *et al*, 2011; Razis *et al*, 2011). Prior experimental studies with SKBR3 and BT474 BC cells and in breast tumour xenografts demonstrated that PTEN reduction confers resistance to trastuzumab's antitumour function, and this data was subsequently confirmed in a group of patients (Nagata *et al*, 2004). In the current study, PTEN loss or promoter hypermethylation were observed both in 20% of the tumours but without association with patient's survival, despite their correlation with other adverse clinicopathological data, such as vascular invasion and lymph node metastases. Nevertheless, tumours with *PTEN* mutations (26%) recurred more frequently in patients with metastatic disease, supporting its contribution to trastuzumab resistance.

The PI3K/Akt pathway activation blocks apoptosis and promotes cellular proliferation through interaction with different downstream effectors (Stemke-Hale *et al*, 2008; Di Cosimo and Baselga, 2008; Nahta and O’Regan, 2011; Margariti *et al*, 2011). *PIK3CA* activating mutations, clustered in exons 9 (helical domain) and 20 (kinase domain) have been reported in 18–40% BC, occasionally associated with HER2 phenotype (Stemke-Hale *et al*, 2008) and tumour recurrence (Razis *et al*, 2011). We found *PIK3CA* mutations in 17% of the tumours, unrelated with trastuzumab clinical benefit. In contrast, p110*α* overexpression (19%) had an independent poor prognostic value for progression in patients with advanced stage. Moreover, active Akt in 28% of our tumours, correlated with recurrence and poor patients’ survival, supporting that activation of this pathway contributes to tumour growth and therefore to trastuzumab resistance. Further, inactive Bad seen in 22% of the tumours in association with adverse prognostic parameters, such as high tumour grade, high mitotic index and vascular invasion, predicted shorter survival as a result of non-response, in early stage patients. In partial agreement with our data, Esteva *et al* (2011), in a previous series of 137 metastatic BC, found that PI3K pathway activation (defined as PTEN loss and/or *PIK3CA* mutation) significantly contributed to worse response to trastuzumab and shorter OS. Moreover, pAkt and PTEN status combination showed more power than PTEN loss alone.

mTOR is a key regulator of multiple cell stimuli integrating growth factor and cytokine signals. *In vitro* studies and recent clinical data have confirmed a relationship between mTOR and HER2 (Morrow *et al*, 2011) as well as its role in trastuzumab resistance (Nahta and O’Regan, 2011). In our series, 23% of predominantly pleomorphic tumours contained increased mTOR, and these patients had lymph-node metastasis. Of note, we found that mTOR modulation by PI3K/Akt-dependent mechanisms reflected by its positive correlation with p110*α* and Bad is influenced by IGF1R. Further, supporting its involvement in the mechanisms of trastuzumab responsiveness, all our patients from the group B and negative mTOR tumours were alive at the last follow-up compared with only 77% for those with positive tumours. This is of interest as preclinical models have shown that dual inhibition of both IGF1R – with either monoclonal antibodies or tyrosine kinase inhibitors – and mTOR results in a superior antiproliferative effect over each single strategy, and this combination is now under evaluation in phase I/II trials in patients with BC (Di Cosimo and Baselga, 2008). Nevertheless, the mechanisms of how mTOR inhibitors reverse resistance to trastuzumab still remain unexplained (Nahta and O’Regan, 2011).

The antiproliferative effect of trastuzumab has been also linked to p27, cyclin E-CDK complex or IGF1R interaction in clinical and *in vitro* studies (Lane *et al*, 2001; Lu *et al*, 2004; Nahta *et al*, 2004). Nevertheless, we were not able to demonstrate any significant correlation between p27, biomarkers or clinicopathological data.

MAPK/ERK pathway stimulation has been related with the oncogenic potential of HER2 and trastuzumab resistance (Berns *et al*, 2007; Yao *et al*, 2009). Nevertheless, recent data support the assertion that trastuzumab has less effect on this cell cycle kinetics pathway (Dave *et al*, 2011), and therefore, not relevant in the development of resistance, in line with our study.

Using a full-length MUC1 antibody, we found expression in 80% of the tumours, with neither correlation with prognostic indicators nor survival. Recent studies have now shown it is not related to tumour growth but it is present in newly differentiated stem cells. However, the cleaved form of the MUC1 protein (MUC1^*^) has growth factor receptor-like activity on tumour cells and is detected in populations of pluripotent stem cells (Hikita *et al*, 2008; Mahanta *et al*, 2008). Fessler *et al* (2009) showed upregulation of MUC1^*^ in HER2-amplified/trastuzumab-resistant BC cells. Further treatment with MUC1^*^ antagonists in addition to trastuzumab reversed that resistance (Fessler *et al*, 2009). Therefore, further studies on MUC1^*^ are needed to confirm the previous data in clinical series. Finally, p53 and Ki67, two well-known prognostic factors (Jansen *et al*, 1998; Yamashita *et al*, 2004) that were increased in a significant number of tumours and associated with poor pathological features, lacked significance with respect to survival.

The results reported here mostly agree with those reported in the literature derived from experimental and clinical series. Nevertheless, our study was not conducted in patients included in a clinical trial, and a control (no-trastuzumab) group was not included. Therefore, the additional influence of CT and poor prognostic factors to the markers associated with trastuzumab resistance cannot be stated with certainty.

In summary, we found in about one-forth of HER2 tumours at least one molecular alteration in the PI3K pathway and/or its upstream or downstream effectors. Our data support the complex interactions between EGFR, IGF1R, and the PTEN/PI3K/Akt/Bad and mTOR signalling pathway, which in turn are potentially related with the mechanisms of trastuzumab response. Nevertheless, some of these biomarkers need to be further validated in larger series and introduced into the clinical practice to carefully select patients on the basis of tumour molecular alterations. This is of relevance as novel combinations for targeting simultaneously several factors might suggest another strategy to overcome trastuzumab resistance and enhance response rates.

## Figures and Tables

**Table 1:** Panel of antibodies for the immunohistochemical analysis

**Antibody**	**Clone**	**Dilution**	**Supplier**	**Pretreatment**
ER	6F11	1 : 40	Novocastra (Newcastle, UK)	Citrate buffer pH 6. Autoclave, 8 min
PR	16	1 : 200	Novocastra (Newcastle, UK)	Citrate buffer pH 6. Autoclave, 8 min
*α*IGF1R	24–31	1 : 200	Neomarkers (Freemont, CA, USA)	Citrate buffer pH 6. Autoclave, 8 min
PTEN	6H2.1	1 : 50	Cascade Biosciences (Winchester, MA, USA)	Citrate buffer pH 6. Autoclave, 40 min
p110*α*	Rabbit polyclonal	1 : 25	Cell Signaling (Beverly, MA, USA)	EDTA buffer pH 8. Autoclave, 8 min
pAkt	Rabbit monoclonal	1 : 50	Cell Signaling (Beverly, MA, USA)	EDTA buffer pH 8. Autoclave, 8 min
pBAD	Sc-12969-R	1 : 40	Santa Cruz (Santa Cruz, CA, USA)	EDTA buffer pH 8. Autoclave, 8 min
mTOR	Rabbit polyclonal	1 : 50	Cell Signaling (Beverly, MA, USA)	EDTA buffer pH 8. Autoclave, 8 min
MUC1	BC-2	1 : 40	Signet (Dedham, MA, USA)	Citrate buffer pH 6. Autoclave, 8 min
pMAPK	Rabbit IgG monoclonal	1 : 100	Cell Signaling (Beverly, MA, USA)	EDTA buffer pH 8. Autoclave, 8 min
Ki67	MIB-1	Prediluted	Dako (Carpinteria, CA, USA)	Citrate buffer pH 6. Autoclave, 8 min
p53	DO-7	Prediluted	Dako (Carpinteria, CA, USA)	Citrate buffer pH 6. Autoclave, 8 min
p27	SX53G8	1 : 50	Dako (Carpinteria, CA, USA)	EDTA buffer pH 8. Autoclave, 8 min

**Table 2:** Summary of the main clinicopathological data

	**All cases (*n*=155)**	**Trastuzumab in the metastatic disease (*n*=75)**	**Trastuzumab in the first-line treatment (*n*=67)**
Age (median and range)	55 years (31–92 years)	59 years (31–92 years)	54 years (33–88 years)
Tumour size (median and range)	2.5 cm (1–20 cm)	2.8 cm (1–11 cm)	2.4 cm (4–20 cm)
			
*BC subgroups*			
HER2+/HR+	67	35	29
HER2+/HR−	78	33	37
Unknown	10	7	1
			
*Lymph node status*			
Negative	46	20	24
Positive	89	47	36
Unknown	20	8	7
			
*Stage*			
IA	17	8	8
IIA	29	10	17
IIB	15	8	6
IIIB	42	22	17
IIIA	18	13	5
IIIC	12	9	3
IV	13	3	7
Unknown	9	2	4
			
*Histological grade*			
1	7	1	4
2	50	24	23
3	98	50	40
			
*DCIS*			
<25%	25	12	12
>25%	22	9	13
			
*Follow-up*			
NED	56	5	50
AWD	31	21	10
DOD	57	49	7
LFU	11	0	0

Abbreviations: AWD=alive with disease; BC=breast carcinoma; DCIS=ductal carcinoma *in situ*; DOD=dead of the disease; HR=hormonal receptors; LFU=lost of follow-up; NED=no evidence of disease.

**Table 3:** Statistical correlations between clinicopathological, immunohistochemical and molecular data for all tumours

	**Histological grade**	**Ductal form**	**Nuclear grade**	**Mitosis**	**Lymph node +**	**Vascular invasion**
HR−	0.080^a^	0.024^a^	NS	0.091^a^	NS	NS
EGFR+	0.061	0.083	NS	0.013	0.088	NS
*α*IGF1R+	0.001	NS	0.07	0.004	NS	0.005
PTEN loss	0.065	NS	NS	NS	NS	0.047
*PIK3CA* mut	NS	NS	0.043	NS	NS	NS
pAkt+	NS	NS	NS	NS	NS	NS
pBad+	0.001	NS	0.008	0.002	NS	0.006
mTOR+	NS	NS	0.034	NS	0.12	NS
MAPK+	0.029^a^	NS	NS	NS	NS	NS
Ki67 >20%	0.087	NS	NS	0.021	NS	0.082
p53 >10%	NS	NS	0.009	0.076	NS	NS
p27+(nuclear)	NS	NS	NS	NS	NS	NS

Abbreviations: EGFR=epidermal growth factor 1-receptor; HR=hormonal receptors; IGF1R=insulin-like growth factor 1-receptor; MAPK=mitogen-activated protein kinase; NS=non-significant; PTEN=phosphatase and tensin homologue.

a Inverse relationship.

**Table 4:** Statistical significance according to metastatic site for all patients. (Note: EGFR, p53, p27, and MAPK expression levels were unrelated with metastases)

	**Liver**	**Bone**	**CNS**	**Skin and soft tissue**	**Lung**	**Pleura**	**Lymph nodes**
HR+	NS	0.008	NS	0.069^a^	NS	NS	NS
ER+	NS	0.004	NS	0.082	NS	NS	NS
PR+	NS	0.044	NS	0.090	NS	NS	NS
*α*IGF1R+	0.009^a^	0.031^a^	NS	NS	0.002^a^	N.S.	0.007^a^
							
*PTEN*							
− Loss expr	NS	NS	0.058	NS	NS	NS	NS
− Mutat	NS	NS	NS	0.065	NS	0.099	NS
							
p110*α*	NS	NS	0.0029	NS	NS	NS	NS
							
*PIK3CA*							
− Mutat	NS	NS	NS	NS	0.087	NS	NS
							
pAkt+	NS	0.085	NS	NS	NS	NS	NS
mTOR+	0.069	NS	NS	NS	NS	NS	NS
Ki67 >20%	0.011	0.011	0.037	NS	NS	0.049	0.096
pBad+	0.068	NS	NS	NS	NS	NS	NS

Abbreviations: CNS=central nervous system; ER=oestrogen receptor; HR=hormonal receptors; IHC=immunohistochemistry; mutat=mutations; NS=non-significant; PR=progesterone receptor.

a Inverse relationship.

**Table 5:** Multivariate analysis of histological and biological factors for patients with trastuzumab treatment in the metastatic disease (group A) (Cox model)

**Variables**	***ß***	**Hazard ratio**	**95% CI**	***P*-value**
*Disease-free survival*				
CNS metastasis	1.128	3.59	1.23–10.51	0.020
p110*α*	1.269	2.75	1.14–6.49	0.024
				
*Overall survival*				
Vascular invasion	1.17	3.36	1.22–8.94	0.015
CNS metastasis	1.406	4.22	1.44–12.38	0.009
EGFR	1.630	5.25	1.32–20.92	0.019

Abbreviations: CNS=central nervous system; EGFR=epidermal growth factor 1-receptor.
