# Supplementary figures and images for: Increased signalling of EGFR and IGF1R, and deregulation of PTEN/PI3K/Akt pathway are related with trastuzumab resistance in HER2 breast carcinomas
Source: Br J Cancer. 2012 Mar 27;106(8):1367–73. doi: 10.1038/bjc.2012.85 (PMC3326683; doi:10.1038/bjc.2012.85)

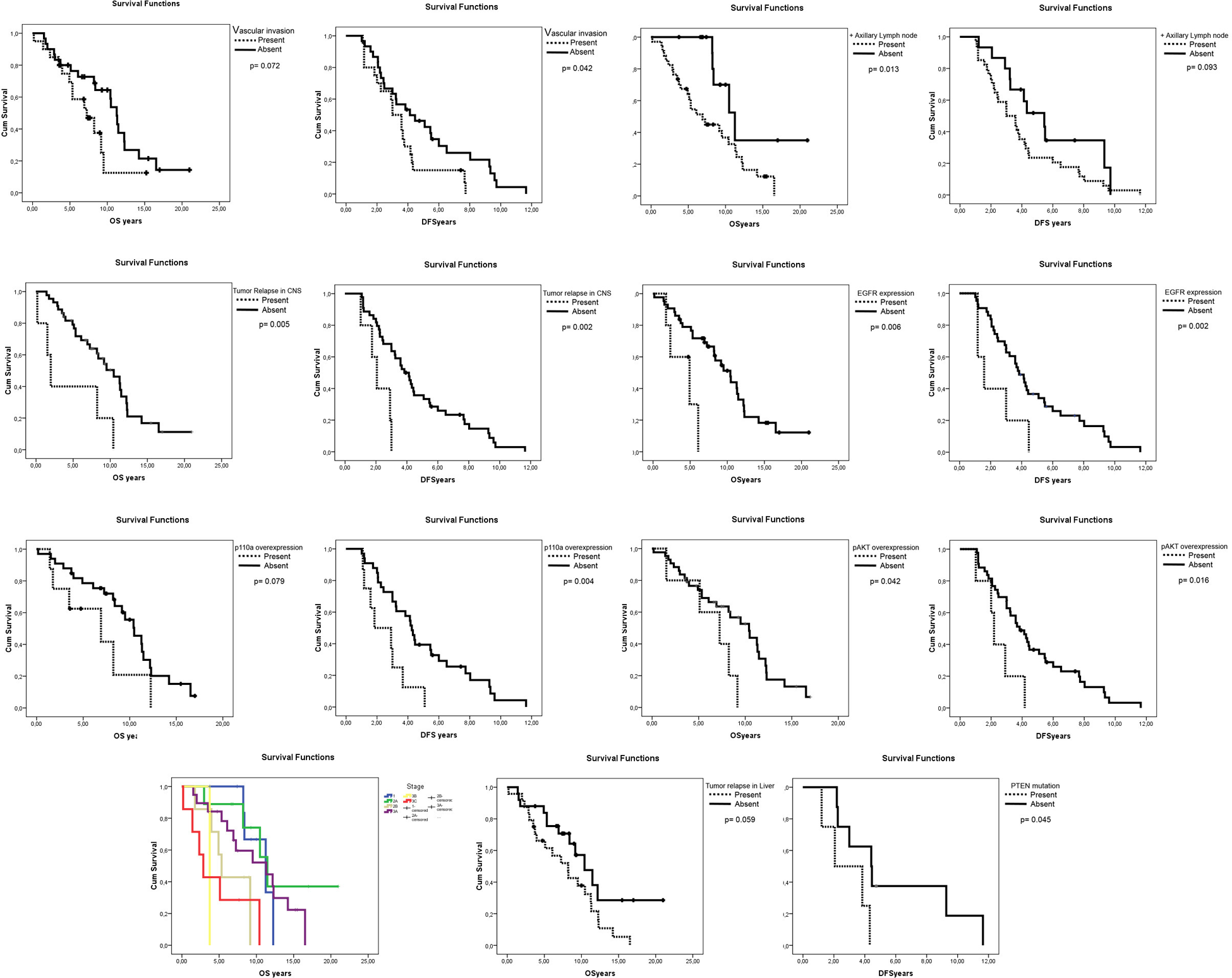

Supplement: Supplementary Figure 1 [file bjc201285x1.tif]

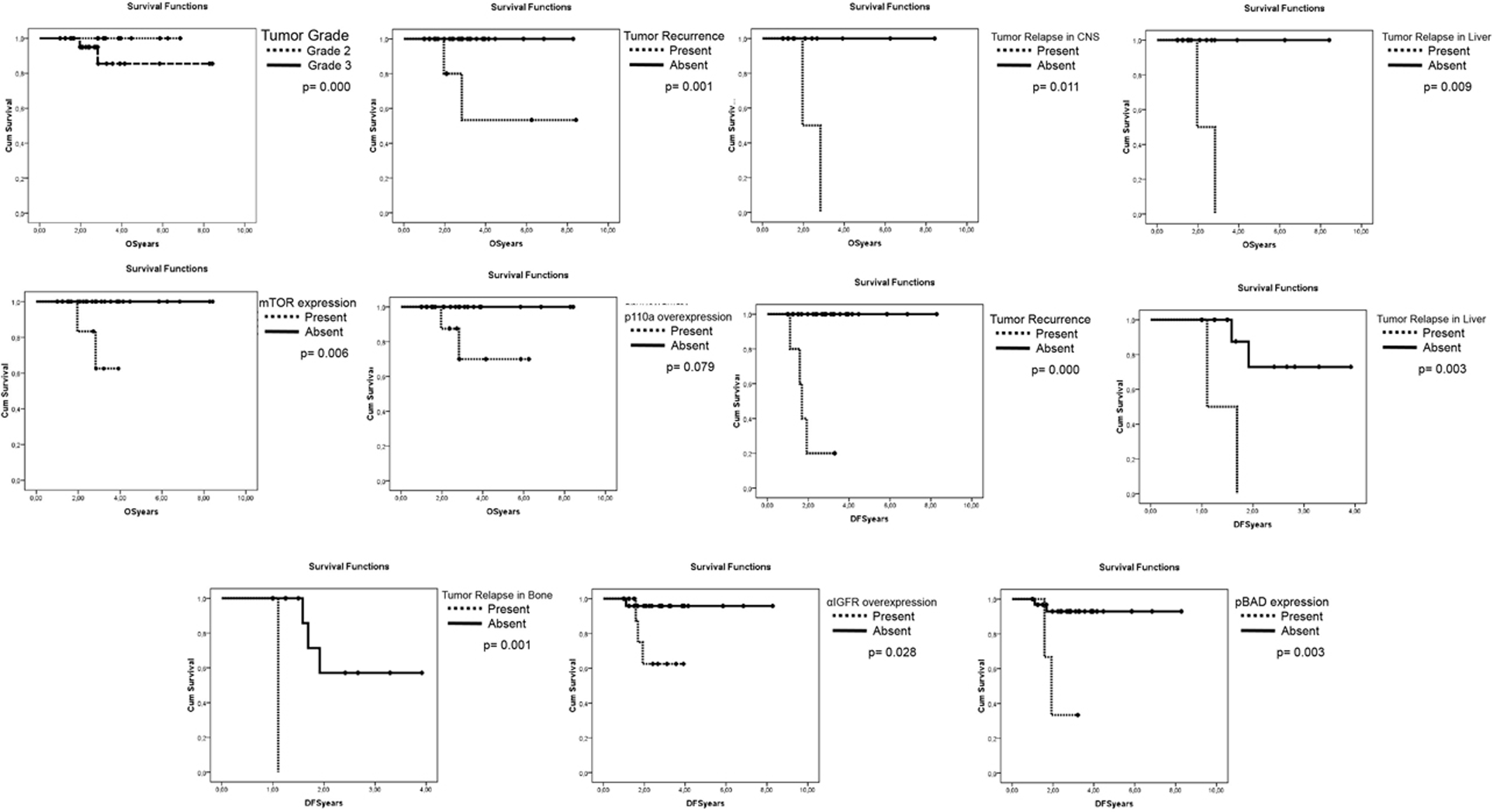

Supplement: Supplementary Figure 2 [file bjc201285x2.tif]
